# Unusual Presentation of Patent Ductus Arteriosus in Elderly Patient

**DOI:** 10.21470/1678-9741-2019-0124

**Published:** 2020

**Authors:** Slobodan V. Micovic, Ivan M. Nesic, Miroslav D. Milicic, Petar M. Vukovic

**Affiliations:** 1 Cardiac Surgery Department, Dedinje Cardiovascular Institute, Belgrade, Serbia.; 2 Medical Faculty University of Belgrade, Dr Subotica 8, Belgrade, Serbia.

**Keywords:** Patent Ductus Arteriosus, Adult Patient, Unusual Localization, Diagnostics

## Abstract

We presented a case of a 56-year-old man with giant pulmonary artery aneurysm caused by a misdiagnosed patent ductus arteriosus, severe multivalvular disease and active aortic valve endocarditis successfully treated by surgery. The correct diagnosis was missed despite preoperative diagnostics because the small patent ductus arteriosus was located at the distal part of common pulmonary trunk and a huge regurgitant signal overlapped its Doppler signal. Thorough evaluation of every patient, regardless of age, is necessary to recognize and treat this congenital anomaly.

**Table:** 

Abbreviations, acronyms & symbols	
MSCT	= Multislice computed tomography
NYHA	= New York Heart Association
PDA	= Patent ductus arteriosus
TEE	= Transesophageal echocardiography
TTE	= Transthoracic echocardiography

## INTRODUCTION

Patent ductus arteriosus (PDA) in adults is an extremely rare clinical phenomenon since it is usually discovered and treated during childhood. However, physicians need to be aware of potential situations, signs and symptoms that might suggest a previously undiagnosed PDA^[1-3]^.

## CASE REPORT

A 56-year-old man was admitted to our institution diagnosed with acute infective endocarditis on aortic valve and severe symptoms of heart failure (NYHA functional class IV), chronic renal failure and sideropenic anemia. He complained on sweating and mild fatigue over the past 20 days, with significant deterioration of his condition in the last four days (febricity up to 38°C, leg edema and loss of breath). He denied having any prior diagnostic and therapeutic invasive procedures as well as other chronic conditions.

The patient lived in a suburban household with his wife. He worked as a locksmith in his own store and did not complain on major limitations in professional or other daily activities related to his condition. The patient rarely drank alcohol and never used drugs. He smoked 15 cigarettes a day for 24 years but quit smoking 11 years ago. He was unable to precise how or where he could have contracted infective endocarditis.

Upon thorough clinical examination, patient’s peripheral venous circulation was adequate, and no evidence of deep vein thrombosis was diagnosed on ultrasonography. Moreover, no ulcerous lesions or varicosities on lower extremities were registered. Laboratory biochemical analyses confirmed renal failure (creatinine 140 µmol/L; urea 11 mmol/L), but liver function was regular (aspartate aminotransferase 15 IU/L; alanine aminotransferase 37 IU/L; lactate dehydrogenase 464 IU/L; prothrombin time 1.14 INR) as well as electrolytic status. All preoperative tests for sexually transmitted diseases were negative (hepatitis B and C, syphilis and HIV).

**Fig. 1:**
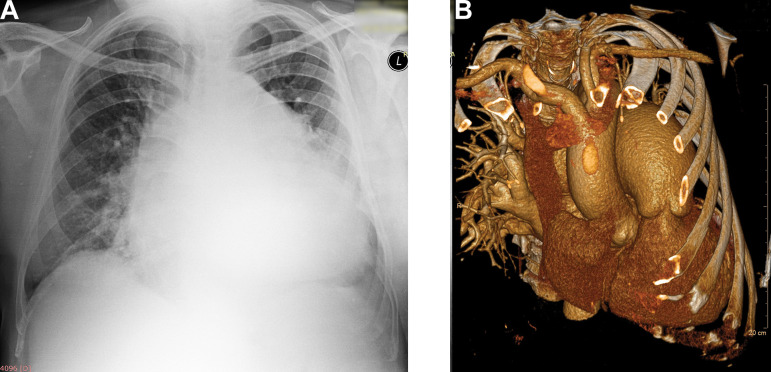
A) preoperative radiography; B) preoperative MSCT.

Preoperative chest radiography revealed extreme cardiomegaly (Figure 1A). Transthoracic and transesophageal echocardiography (TTE and TEE) showed severe aortic regurgitation with the presence of mobile ribbon-like formation with high thromboembolic potential. The aortic root, as measured on TTE, was 26 mm in diameter. Moreover, severe mitral regurgitation, severe pulmonary regurgitation with giant aneurysm of pulmonary trunk, moderate tricuspid regurgitation (right ventricular systolic pressure of 80 mmHg), dilated left atrium and ventricle, with preserved ejection fraction were found. Multislice computed tomography (MSCT) confirmed the presence of pulmonary artery aneurysm (77.8 mm in diameter) (Figure 1B).

**Fig. 2:**
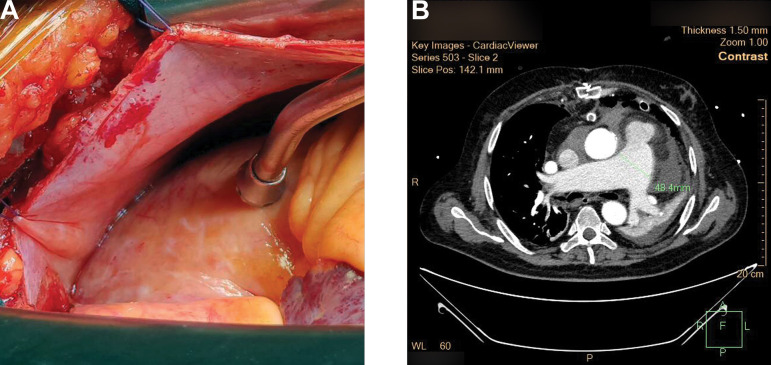
A) intraoperative findings; B) postoperative MSCT.

Upon these numerous conditions, heart surgery was indicated and performed through the median sternotomy. Cardiopulmonary bypass was initiated using central aortic and bicaval venous cannulation. The interatrial approach (through the Sondergaard's groove) was used to expose the mitral valve. Mitral leaflets were normal without endocardial thickening, calcifications, vegetations, thrombus formations or other pathological findings. The subvalvular apparatus was preserved without chordal thickening, chordal rupture or elongation and the papillary muscles without fibrosis or other abnormalities. Mitral annulus was dilated. Downsizing annuloplasty was performed with the Physio-Ring size 30. Aortic valve replacement with mechanical valve was performed. Intraoperative measurements of aortic annulus indicated the placement of a 23 mm artificial valve. During the operation, due to excessive blood inflow to the heart through the pulmonary trunk (Figure 2A), suspicions about the existence of PDA were raised. Consequently, the pulmonary trunk was opened through longitudinal incision of the anterior portion, which confirmed the presence of PDA. The PDA (4-5 mm in diameter) was positioned in the more proximal and inferior location than usual. The duct was closed with three pledgeted polypropylene sutures.

Pulmonary valve was regurgitant due to enlargement of the annulus and main pulmonary trunk. Leaflets were stretched and fibrously replaced with suspicious small vegetation on the ventricular side of the valve. Decision was made to replace the valve using bioprosthetic St. Jude Epic valve size 27. Pulmonary trunk diameter was reduced by generous longitudinal excision of the anterior portion. It was reconstructed by running suture with buttressing strips of bovine pericardium.

Weaning from cardiopulmonary bypass was uneventful. Intraoperative TEE was normal. Mechanical ventilation was prolonged, and weaning was done on the 2^nd^ postoperative day, but it was spontaneous, without need for any medications or interventions.

Postoperatively, our patient was treated with supportive, symptomatic anticoagulant and other cardiologic therapy. As *Staphylococcus aureus* was cultivated in aortic tissue culture, he also received antibiotic therapy (vancomycin) postoperatively. Postoperative TTE after seven days and MSCT after three days revealed normal findings and reduced pulmonary trunk diameter to around 45 mm (Figure 2B). Moderate postoperative respiratory insufficiency due to left-sided atelectasis was successfully treated by bronchoscopy and intensive respiratory rehabilitation. On the 10^th^ postoperative day, the patient was transferred to the secondary referral center for further treatment until full recovery.

## DISCUSSION

PDA accounts for 5-12% of all congenital heart defects and is found twice as often in females. Mortality of untreated PDA in adults is estimated at 1.8% per year^[2,4]^. In adults, PDA is usually incidentally discovered during physical examination or echocardiographic screening. PDA typically presents a continuous murmur heard at the upper left sternal border, but may be completely asymptomatic^[3,4]^. Silent PDA, tolerated for years, may become clinically significant when acquired conditions such as the development of chronic obstructive pulmonary disease, or manifestations of valvular or ischemic heart disease are present^[2-4]^. Some patients can develop congestive heart failure, pulmonary hypertension, signs of right or left heart volume overload, endocarditis, or recurrent pneumonia. Pulmonary artery aneurysms diagnosed in our patient, also a rare condition, could also be a consequence of structural cardiac anomalies, especially congenital heart diseases such as PDA^[2-4]^.

Literature data shows that TEE is more sensitive in detecting adult PDA than TTE. Diagnosis can then be confirmed by MSCT or magnetic resonance imaging^[1]^. A variety of surgical repair techniques are used, mainly influenced by the size, shape, calcification and friability of the PDA^[5]^.

Our patient had an extraordinary giant pulmonary artery aneurysm caused by misdiagnosed PDA, severe multivalvular disease and active aortic valve endocarditis.

Severity of valvular disease, mitral, aortic, and pulmonary insufficiency misled physicians in diagnosis despite performing both TEE and MSCT. TEE was not useful because the small duct was unusually located and a huge regurgitant signal overlapped its Doppler signal. The presence of a giant aneurysm made the TEE examination difficult and prevented proper cardiac evaluation. Moreover, MSCT scan was performed without contrast, as the patient had severe renal failure and was in very poor condition.

The incidence of unrecognized PDA in adult populations is still high, even with current diagnoses. Its different clinical presentations can easily mislead physicians. If excessive blood inflow to the heart through the pulmonary trunk aneurysm appears during surgery, unrecognized PDA should be suspected.

**Table:** 

Author's roles & responsibilities	
SVM	Substantial contributions to the conception or design of the work; or the acquisition, analysis, or interpretation of data for the work; drafting the work or revising it critically for important intellectual content; final approval of the version to be published
IMN	Substantial contributions to the conception or design of the work; or the acquisition, analysis, or interpretation of data for the work; drafting the work or revising it critically for important intellectual content; final approval of the version to be published
MDM	Substantial contributions to the conception or design of the work; or the acquisition, analysis, or interpretation of data for the work; drafting the work or revising it critically for important intellectual content; final approval of the version to be published
PMV	Substantial contributions to the conception or design of the work; or the acquisition, analysis, or interpretation of data for the work; drafting the work or revising it critically for important intellectual content; final approval of the version to be published
